# Supplementary figures and images for: Characterization of geographic mobility among participants in facility- and community-based tuberculosis case finding in urban Uganda
Source: PLoS One. 2021 May 14;16(5):e0251806. doi: 10.1371/journal.pone.0251806 (PMC8121348; doi:10.1371/journal.pone.0251806)

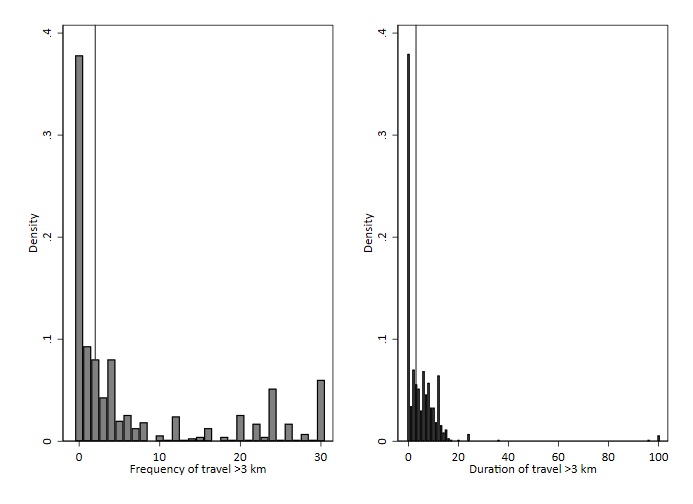

Supplement: S1 Fig — (PNG) [file pone.0251806.s001.png]
