# Supplementary material for: Characterization of geographic mobility among participants in facility- and community-based tuberculosis case finding in urban Uganda
Source: PLoS One. 2021 May 14;16(5):e0251806. doi: 10.1371/journal.pone.0251806 (PMC8121348; doi:10.1371/journal.pone.0251806)
Supplement: S1 Table — (DOCX) [file pone.0251806.s002.docx]

**Table S3. Model selection**

| **Number of Latent Classes** | **Degrees of freedom** | **Bayesian Information Criterion** |
| --- | --- | --- |
| 1 | 8 | 5922.9 |
| 2 | 17 | 5552.7 |
| 3 | 26 | 5563.1 |
| 4 | 35 | 5597.0 |
