# Supplementary material for: Characterization of geographic mobility among participants in facility- and community-based tuberculosis case finding in urban Uganda
Source: PLoS One. 2021 May 14;16(5):e0251806. doi: 10.1371/journal.pone.0251806 (PMC8121348; doi:10.1371/journal.pone.0251806)
Supplement: S2 Table — (DOCX) [file pone.0251806.s003.docx]

**Table S4. Estimated Marginal Means for Latent Classes of Mobility**

|  | **Class 1**  **(Mobile)**  **Mean (95%CI)** | **Class 2**  **(Non-mobile)**  **Mean (95%CI)** | **Difference***  **(Class 1 – Class 2)** |
| --- | --- | --- | --- |
| **Marginal probability of class membership** | **0.51 (0.45-0.57)** | **0.49 (0.43-0.55)** | **---** |
| Travel 3km ≥2 times per month | 1 (0-1) | 0.04 (0-0.35) | 0.96 |
| Spend ≥3 hours away when traveling 3km | 0.87 (0.78-0.92) | 0.15 (0.11-0.19) | 0.72 |
| Visits taxi stage ≥1 time per week | 0.36 (0.31-0.41) | 0.18 (0.14-0.22) | 0.18 |
| Lived in neighborhood <1 year | 0.14 (0.11-0.18) | 0.25 (0.2-0.3) | -0.11 |
| Traveled outside Kampala in last year | 0.81 (0.76-0.85) | 0.71 (0.66-0.75) | 0.10 |
| Spends ≥10 nights away from primary residence | 0.14 (0.11-0.18) | 0.06 (0.04-0.1) | 0.08 |
| Have another residence | 0.15 (0.11-0.19) | 0.16 (0.12-0.2) | -0.01 |
| Born outside Kampala | 0.84 (0.79-0.87) | 0.84 (0.79-0.87) | 0 |

*sorted by magnitude
