# Supplementary material for: Characterization of geographic mobility among participants in facility- and community-based tuberculosis case finding in urban Uganda
Source: PLoS One. 2021 May 14;16(5):e0251806. doi: 10.1371/journal.pone.0251806 (PMC8121348; doi:10.1371/journal.pone.0251806)
Supplement: S3 Table — (DOCX) [file pone.0251806.s004.docx]

**Table S5. Estimated Marginal Means for Latent Classes of Mobility stratified by case status**

|  | **All cases** | | | **All controls** | | |
| --- | --- | --- | --- | --- | --- | --- |
|  | **Class 1**  **(Mobile)**  **Mean (95%CI)** | **Class 2**  **(Non-mobile)**  **Mean (95%CI)** | **Difference***  **(Class 1 – Class 2)** | **Class 1**  **(Mobile)**  **Mean (95%CI)** | **Class 2**  **(Non-mobile)**  **Mean (95%CI)** | **Difference***  **(Class 1 – Class 2)** |
| **Marginal probability of class membership** | **0.48 (0.40-0.57)** | **0.52 (0.43-0.60)** | **---** | **0.52 (0.45-0.59)** | **0.48 (0.41-0.55)** | **---** |
| Travel 3km ≥2 times per month | 1 (0-1) | 0.05 (0-0.39) | 0.95 | 1 (0-1) | 0.05 (0-0.36) | 0.95 |
| Spend ≥3 hours away when traveling 3km | 0.93 (0.73-0.99) | 0.15 (0.10-0.23) | 0.78 | 0.84 (0.74-0.91) | 0.15 (0.1-0.2) | 0.69 |
| Visits taxi stage ≥1 time per week | 0.33 (0.26-0.42) | 0.19 (0.13-0.26) | 0.14 | 0.37 (0.31-0.44) | 0.17 (0.12-0.23) | 0.20 |
| Lived in neighborhood <1 year | 0.16 (0.10-0.23) | 0.21 (0.15-0.29) | -0.05 | 0.13 (0.09-0.18) | 0.27 (0.21-0.34) | -0.14 |
| Traveled outside Kampala in last year | 0.80 (0.72-0.87) | 0.69 (0.60-0.76) | 0.11 | 0.82 (0.76-0.86) | 0.72 (0.65-0.78) | 0.10 |
| Spends ≥10 nights away from primary residence | 0.12 (0.07-0.19) | 0.04 (0.02-0.09) | 0.08 | 0.15 (0.11-0.21) | 0.08 (0.05-0.13) | 0.07 |
| Have another residence | 0.13 (0.08-0.02) | 0.11 (0.07-0.18) | 0.02 | 0.16 (0.12-0.21) | 0.19 (0.14-0.25) | -0.03 |
| Born outside Kampala | 0.75 (0.66-0.82) | 0.79 (0.71-0.85) | -0.04 | 0.89 (0.84-0.92) | 0.86 (0.81-0.9) | -0.03 |
