# Supplementary material for: Characterization of geographic mobility among participants in facility- and community-based tuberculosis case finding in urban Uganda
Source: PLoS One. 2021 May 14;16(5):e0251806. doi: 10.1371/journal.pone.0251806 (PMC8121348; doi:10.1371/journal.pone.0251806)
Supplement: S4 Table — (DOCX) [file pone.0251806.s005.docx]

**Table S6. Estimated Marginal Means for Latent Classes of Mobility stratified by enrollment method**

|  | **Health facility enrollment** | | | **Community case finding enrollment** | | |
| --- | --- | --- | --- | --- | --- | --- |
|  | **Class 1**  **(Mobile)**  **Mean (95%CI)** | **Class 2**  **(Non-mobile)**  **Mean (95%CI)** | **Difference***  **(Class 1 – Class 2)** | **Class 1**  **(Mobile)**  **Mean (95%CI)** | **Class 2**  **(Non-mobile)**  **Mean (95%CI)** | **Difference***  **(Class 1 – Class 2)** |
| **Marginal probability of class membership** | **0.50 (0.42-0.58)** | **0.50 (0.42-0.58)** | **---** | **0.59 (0.49-0.70)** | **0.41 (0.30-0.51)** | **---** |
| Travel 3km ≥2 times per month | 1 (0-1) | 0 (0-1) | 1.0 | 0.94 (0.78-0.98) | 0.09 (0.02-0.35) | 0.85 |
| Spend≥3 hours away when traveling 3km | 0.85 (0.73-0.93) | 0.16 (0.12-0.21) | 0.69 | 0.87 (0.73-0.95) | 0.04 (0-0.43) | 0.83 |
| Visits taxi stage ≥1 time per week | 0.22 (0.18-0.28) | 0.12 (0.09-0.17) | 0.10 | 0.62 (0.52-0.71) | 0.33 (0.23-0.45) | 0.29 |
| Lived in neighborhood <1 year | 0.13 (0.09-0.18) | 0.25 (0.20-0.31) | -0.08 | 0.16 (0.10-0.24) | 0.25 (0.16-0.37) | -0.09 |
| Traveled outside Kampala in last year | 0.79 (0.73-0.84) | 0.71 (0.65-0.76) | 0.08 | 0.86 (0.78-0.91) | 0.69 (0.57-0.78) | 0.17 |
| Spends ≥10 nights away from primary residence | 0.12 (0.08-0.17) | 0.06 (0.03-0.09) | 0.06 | 0.17 (0.11-0.25) | 0.09 (0.04-0.17) | 0.08 |
| Have another residence | 0.12 (0.09-0.17) | 0.15 (0.11-0.20) | -0.03 | 0.20 (0.14-0.29) | 0.17 (0.10-0.27) | 0.03 |
| Born outside Kampala | 0.84 (0.79-0.88) | 0.82 (0.77-0.86) | -0.02 | 0.80 (0.72-0.87) | 0.92 (0.82-0.96) | -0.12 |
