# Supplementary material for: Characterization of geographic mobility among participants in facility- and community-based tuberculosis case finding in urban Uganda
Source: PLoS One. 2021 May 14;16(5):e0251806. doi: 10.1371/journal.pone.0251806 (PMC8121348; doi:10.1371/journal.pone.0251806)
Supplement: S5 Table — (DOCX) [file pone.0251806.s006.docx]

**Table S7. Estimated Marginal Means for Latent Classes of Mobility - sensitivity analysis using Poisson-distributed variables**

|  |  | **Sensitivity Analysis 1** | | | **Sensitivity Analysis 2** | | |
| --- | --- | --- | --- | --- | --- | --- | --- |
|  | **Class 1**  **(Mobile)**  **Mean (95%CI)** |  | **Class 2**  **(Non-mobile)**  **Mean (95%CI)** | **Difference***  **(Class 1 – Class 2)** | **Class 1**  **(Mobile)**  **Mean (95%CI)** | **Class 2**  **(Non-mobile)**  **Mean (95%CI)** | **Difference***  **(Class 1 – Class 2)** |
| **Marginal probability of class membership** | **0.26 (0.23-0.30)** | | **0.74 (0.70-0.77)** | **---** | **0.51 (0.46-0.55)** | **0.49 (0.45-0.54)** | **---** |
| Travel >3km ≥2 times per month | --- | | --- | --- | 0.90 (0.86-0.93) | 0.15 (0.10-0.21) | 0.75 |
| Frequency of travel >3km per month (Poisson-distributed) | 21.9 (21.1-22.6) | | 1.47 (1.35-1.58) | 20.43 | --- | --- | --- |
| Spend ≥3 hours away when traveling >3km | 0.88 (0.82-0.92) | | 0.39 (0.35-0.43) | 0.49 | --- | --- | --- |
| Duration (in hours) of travel >3km (Poisson-distributed) | --- | | --- | --- | 9.46 (8.95-9.97) | 0.45 (0.29-0.60) | 9.01 |
| Visits taxi stage ≥1 time per week | 0.43 (0.36-0.50) | | 0.21 (0.18-0.25) | 0.22 | 0.33 (0.28-0.38) | 0.20 (0.16-0.25) | 0.13 |
| Lived in neighborhood <1 year | 0.16 (0.12-0.22) | | 0.20 (0.17-0.24) | -0.04 | 0.15 (0.11-0.19) | 0.24 (0.20-0.29) | -0.09 |
| Traveled outside Kampala in last year | 0.79 (0.72-0.84) | | 0.75 (0.71-0.79) | 0.04 | 0.81 (0.77-0.85) | 0.71 (0.66-0.75) | 0.10 |
| Spends ≥10 nights away from primary residence | 0.19 (0.14-0.26) | | 0.07 (0.05-0.10) | 0.12 | 0.14 (0.10-0.18) | 0.07 (0.05-0.10) | 0.07 |
| Have another residence | 0.15 (0.10-0.21) | | 0.15 (0.12-0.19) | 0.00 | 0.16 (0.12-0.20) | 0.15 (0.11-0.19) | 0.01 |
| Born outside Kampala | 0.83 (0.78-0.89) | | 0.83 (0.80-0.86) | 0.00 | 0.83 (0.79-0.87) | 0.84 (0.80-0.87) | -0.01 |
