# Supplementary material for: Characterization of geographic mobility among participants in facility- and community-based tuberculosis case finding in urban Uganda
Source: PLoS One. 2021 May 14;16(5):e0251806. doi: 10.1371/journal.pone.0251806 (PMC8121348; doi:10.1371/journal.pone.0251806)
Supplement: S6 Table — (DOCX) [file pone.0251806.s007.docx]

**Table S8. Estimated Marginal Means for Latent Classes of Mobility - sensitivity analysis for dichotomizing continous variables**

|  |  | **Sensitivity Analysis 3** | | | **Sensitivity Analysis 4** | | | **Sensitivity Analysis 5** | | |
| --- | --- | --- | --- | --- | --- | --- | --- | --- | --- | --- |
|  | **Class 1**  **(Mobile)**  **Mean (95%CI)** |  | **Class 2**  **(Non-mobile)**  **Mean (95%CI)** | **Difference***  **(Class 1 – Class 2)** | **Class 1**  **(Mobile)**  **Mean (95%CI)** | **Class 2**  **(Non-mobile)**  **Mean (95%CI)** | **Difference***  **(Class 1 – Class 2)** | **Class 1**  **(Mobile)**  **Mean (95%CI)** | **Class 2**  **(Non-mobile)**  **Mean (95%CI)** | **Difference***  **(Class 1 – Class 2)** |
| **Marginal probability of class membership** | **0.49 ( 0.43-0.55)** |  | **0.51 (0.45-0.57)** | **---** | **0.35 (0.24-0.47)** | **0.65 (0.53-0.76)** | **---** | **0.48 (0.40-0.56)** | **0.52 (0.44-0.60)** | **---** |
| Travel 3km ≥2 times per month | 1 (0-1) |  | 0.07 (0.02-0.23) | 0.93 | --- | --- | --- | 0.94 (0.89-0.96) | 0.15 (0.07-0.3) | 0.78 |
| Travel 3km ≥8 times per month (75th percentile) | --- |  | --- | --- | 0.71 (0.49-0.87) | 0.03 (0.01-0.14) | 0.68 | --- | --- | --- |
| Spend ≥3 hours away when traveling 3km | 0.89 (0.79-0.95) |  | 0.15 (0.11-0.19) | 0.74 | 0.91 (0.78-0.97) | 0.31 (0.22-0.41) | 0.6 | --- | --- | --- |
| Spend ≥8 hours away when traveling 3km (75th percentile) | --- |  | --- | --- | --- | --- | --- | 0.55 (0.45-0.64) | 0 (0-1) | 0.55 |
| Visits taxi stage ≥1 time per week | 0.36 (0.31-0.41) |  | 0.18 (0.14-0.22) | 0.18 | 0.43 (0.35-0.51) | 0.18 (0.14-0.23) | 0.24 | 0.38 (0.33-0.45) | 0.16 (0.12-0.21) | 0.22 |
| Lived in neighborhood <1 year | 0.14 (0.1-0.18) |  | 0.24 (0.2-0.29) | -0.11 | 0.14 (0.09-0.2) | 0.22 (0.18-0.27) | -0.08 | 0.13 (0.1-0.18) | 0.25 (0.2-0.3) | -0.11 |
| Traveled outside Kampala in last year | 0.81 (0.77-0.85) |  | 0.71 (0.66-0.75) | 0.10 | 0.82 (0.75-0.87) | 0.73 (0.68-0.77) | 0.09 | 0.85 (0.79-0.89) | 0.68 (0.63-0.73) | 0.17 |
| Spends ≥10 nights away from primary residence | --- |  | --- | --- | 0.19 (0.13-0.26) | 0.06 (0.04-0.09) | 0.13 | 0.15 (0.11-0.2) | 0.06 (0.04-0.09) | 0.1 |
| Spends ≥1 night away from primary residence | 0.27 (0.22-0.32) |  | 0.11 (0.08-0.14) | 0.16 | --- | --- | --- | --- | --- | --- |
| Have another residence | 0.15 (0.11-0.19) |  | 0.15 (0.12-0.2) | -0.01 | 0.16 (0.11-0.22) | 0.15 (0.12-0.19) | 0.01 | 0.17 (0.13-0.21) | 0.14 (0.1-0.18) | 0.03 |
| Born outside Kampala | 0.84 (0.79-0.87) |  | 0.83 (0.79-0.87) | 0.00 | 0.83 (0.76-0.88) | 0.84 (0.8-0.87) | -0.01 | 0.83 (0.79-0.87) | 0.84 (0.79-0.87) | 0 |
