# Supplementary material for: Characterization of geographic mobility among participants in facility- and community-based tuberculosis case finding in urban Uganda
Source: PLoS One. 2021 May 14;16(5):e0251806. doi: 10.1371/journal.pone.0251806 (PMC8121348; doi:10.1371/journal.pone.0251806)
Supplement: S7 Table — (DOCX) [file pone.0251806.s008.docx]

**Table S9. Mobility and duration of TB related symptoms (prior to enrollment) among symptomatic individuals with TB who reported symptoms**

|  | **Mobile**  (median, IQR) | **Non-mobile**  (median, IQR) | **Difference in medians**  **(95% CI)** | **Wilcoxon Rank-Sum**  **p-value** |
| --- | --- | --- | --- | --- |
| **Duration of TB-related symptoms** |  |  |  |  |
| Health facility enrollment | 8 (4,20) | 8 (4,16) | 0 (-4.5, 4.5) | 0.66 |
| Community enrollment | 4 (2, 16) | 3 (1, 9.5) | -1 (-3.9, 1.9) | 0.30 |
| **Number of symptom-related health care visits** |  |  |  |  |
| Health facility enrollment | 6 (3, 10) | 5 (4,8) | -1 (-2.6, .6) | 0.36 |
| Community enrollment | 2 (0, 5) | 3 (1, 5.5) | 1 (-1.0, 3.0) | 0.24 |
