# Supplementary material for: Characterization of geographic mobility among participants in facility- and community-based tuberculosis case finding in urban Uganda
Source: PLoS One. 2021 May 14;16(5):e0251806. doi: 10.1371/journal.pone.0251806 (PMC8121348; doi:10.1371/journal.pone.0251806)
Supplement: S1 Appendix — (PDF) [file pone.0251806.s009.pdf]

8.4. If you were asked by a community health worker to provide each of the following to help find out the reason for a cough in the future, how likely would you be to agree?

	Would definitely participate	Would possibly participate	Probably would not participate	Certainly would not participate	Unknown/refused
Sputum specimen that you would cough into a cup	◯	◯	◯	◯	◯
A chest X-ray	◯	◯	◯	◯	◯
A blood specimen (by finger prick)	◯	◯	◯	◯	◯
A urine specimen	◯	◯	◯	◯	◯
A skin test , which involves injecting a small amount of fluid just under the skin of your forearm to see if your body reacts to it	◯	◯	◯	◯	◯
Several questions asked to determine if you have any TB symptoms, with advice that you should seek medical care if your answers suggest that you may have TB	◯	◯	◯	◯	◯

8.5. Some people who don't have TB now may be at risk for developing TB in the future. It may be possible to take medicine now to prevent TB in the future. If you did not have TB and you were feeling well, how likely would you be to agree to take medicine of the following types to lower your risk of developing future TB?

	Would definitely participate	Would possibly participate	Probably would not participate	Certainly would not participate	Unknown/refused
One pill plus a vitamin every day for 6 months	◯	◯	◯	◯	◯
9 pills taken at once, once a week for 3 months	◯	◯	◯	◯	◯
a single injection given one time	◯	◯	◯	◯	◯

9.0. Movement and migration

9.1. Where were you born?

◯ Kampala
◯ Central Uganda
◯ Northern Uganda
◯ Eastern Uganda
◯ Western Uganda
◯ Outside Uganda
◯ Unknown

9.1.a. Specify where in Kampala you were born?

◯ Kisugu
◯ Wabigalo
◯ Bukasa
◯ Other
◯ Unknown

9.1b Specify the "other" location of birth within Kampala

____

9.2. Outside Uganda(Specify)

◯ Kenya
◯ Burundi
◯ Tanzania
◯ South Sudan
◯ Rwanda
◯ D.R.C
◯ India
◯ Other
◯ Unknown

Specify the "other" location of birth outside Uganda

____

9.3. How long have you lived at your current residence, in months?
If you currently live in more than one house or address, then please tell us about the place you live within Kisugu, Wabigalo, or Namuwongo if you have such a residence. Otherwise, tell us about the place where you live most of the time. (This should be the same residence that is identified by GPS coordinates above, and that is described in other questions about the participant's residence unless otherwise specified.)

____
(Record approximate number of months, or ''-999'' if unable to estimate.)

9.3.1 Out of 30 nights per month, how many nights do you stay in this residence?

____

9.3.2 On a typical day that you are staying at this house, how many hours (out of 24 hours) do you spend inside this house?

____

	Rarely (less than 20% of the time)	Sometimes (20-49% of the time)	Often (50-80% of the time)	Always (more than 80% of the time)
Weekdays at 9:00am	◯	◯	◯	◯
Weekdays at 3:00pm	◯	◯	◯	◯
Weekdays at 7:00pm	◯	◯	◯	◯

9.3.3 I am now going to ask you some questions about how much time you spend in your household at different times. I will ask you about a day of the week and a time during that day. Think about how often you are typically at home at that time (for example, in each week during the last month).

Saturdays at 9:00am	◯	◯	◯	◯
Saturdays at 3:00pm	◯	◯	◯	◯
Saturdays at 7:00pm	◯	◯	◯	◯
Sundays at 9:00am	◯	◯	◯	◯
Sundays at 3:00pm	◯	◯	◯	◯
Sundays at 7:00pm	◯	◯	◯	◯

9.3b Is there another place that you stay, other than the residence you have just described?

◯ No
◯ Yes
◯ Unknown/refused

9.3c How would you describe the other place that you stay?	◯ A permanent residence (>6 months)<br>◯ A transient residence (1-6 months)<br>◯ A place you visit (less than 1 month)<br>◯ Unsure/unwilling to answer
9.3d Approximately how far away (in kilometers from the residence you described previously) is this other place that you stay?	__________________________________
9.4. Now think about the last place you lived before you began living in your current residence. Approximately how far in kilometers was that place from your current residence?	__________________________________<br>(Use place examples like Kisugu stage to old Taxi Park, in Kilometers(km))
9.5a. Estimate the total amount of time you have lived in Kampala. If you left Kampala and returned later, include the time before you left.<br><br>(Round to the nearest year.)	__________________________________<br>(If less than 6 months or if never, enter 0.)
9.5b. Estimate the total amount of time you have lived inside the boundaries of Kisugu and/or Wabigalo parishes or the Namuwongo area of Bukasa. If you have lived at different residences in these areas, include time lived at any of them.<br><br>(Round to the nearest year.)	__________________________________<br>(If less than 6 months, enter 0.)
9.6. Our study team previously attempted to test everyone in Kisugu, Wabigalo, and Namuwongo for TB during [~2019]. Did you provide or attempt to provide a sputum specimen as part of this study at that time?	◯ Yes, Provided sputum for testing<br>◯ Attempted but was unable to provide sputum<br>◯ No, did not participate<br>◯ Unsure
9.7. Now I want you to think about what places are within about 3 kilometers from your residence - that is, less than about a 10 minute boda ride or 30 minute brisk walk from where you live. For approximately how long have you lived within about 3 km of your current residence even if you were not living in your current residence)?	__________________________________<br>(Record approximate number of months, or "-999" if unable to estimate.)
9.8. How often (how many times per month) do you go somewhere more than about 3km from your residence?	__________________________________<br>(Record approximate number per month, or "-999" if unable to estimate.)
9.8.1 Since the start of COVID-related lockdowns in March 2020, how often (how many times per month) have you gone somewhere more than about 3km from your residence?	__________________________________<br>(Record approximate number per month, or "-999" if unable to estimate.)
9.9. When you go more than about 3km from your residence, how many hours do you usually spend >3km away?	__________________________________<br>(-999' if unable to estimate)

9.10. What is the most common way you make trips >3 km from your residence?

- ◯ Walking
- ◯ Bicycle
- ◯ Boda-boda/motorbike
- ◯ Mini bus
- ◯ Bus
- ◯ Taxi
- ◯ Private car
- ◯ Other
- ◯ Unknown/not answered

Other (Specify) ______________________________

9.11. How many times did you travel outside of Kampala in the past 12 months, to your best recollection? ______________________________

(If they moved to Kampala within the past 12 months, record trips since moving to Kampala. -999' if unable to estimate. 0 if they never left.)

9.12. What is the place outside Kampala that you visit most often? ______________________________

(If the participant states he/she does not leave Kampala, enter "none" (all lowercase))

9.13. How far away would you estimate that place is, in kilometers? ______________________________

9.14. Now think of the farthest place from your residence that you have been to in the past 12 months. Approximately how many kilometers is that place from your residence? ______________________________

10.0 Note: Health status, depression screening and alcohol screening. Note: these questions have been validated as written; please ask them in this form.

10.1. Over the past two weeks, how often have you been bothered by

	Not at all	Several days	More than half the days	Nearly every day	Unable/unwilling to answer
...little interest or pleasure in doing things?	◯	◯	◯	◯	◯
...feeling down, depressed, or hopeless?	◯	◯	◯	◯	◯

10.2. Now I am going to ask you some questions about your use of alcoholic drinks during this past year (meaning the past 12 months). How often did you have a drink containing alcohol in the past year?

- ◯ Never
- ◯ Monthly or less
- ◯ Two to four times a month
- ◯ Two to three times per week
- ◯ Four or more times a week
- ◯ Refused
